# Cell Reprogramming and Differentiation Utilizing Messenger RNA for Regenerative Medicine

**DOI:** 10.3390/jdb12010001

**Published:** 2023-12-20

**Authors:** Masahito Inagaki

**Affiliations:** Graduate School of Science, Nagoya University, Nagoya 464-8602, Japan; inagaki.masahito.e6@f.mail.nagoya-u.ac.jp

**Keywords:** messenger RNA, cell regeneration, pluripotent cells, cellular differentiation, direct reprogramming, regenerative medicine

## Abstract

The COVID-19 pandemic generated interest in the medicinal applications of messenger RNA (mRNA). It is expected that mRNA will be applied, not only to vaccines, but also to regenerative medicine. The purity of mRNA is important for its medicinal applications. However, the current mRNA synthesis techniques exhibit problems, including the contamination of undesired 5′-uncapped mRNA and double-stranded RNA. Recently, our group developed a completely capped mRNA synthesis technology that contributes to the progress of mRNA research. The introduction of chemically modified nucleosides, such as N1-methylpseudouridine and 5-methylcytidine, has been reported by Karikó and Weissman, opening a path for the practical application of mRNA for vaccines and regenerative medicine. Yamanaka reported the production of induced pluripotent stem cells (iPSCs) by introducing four types of genes using a retrovirus vector. iPSCs are widely used for research on regenerative medicine and the preparation of disease models to screen new drug candidates. Among the Yamanaka factors, Klf4 and c-Myc are oncogenes, and there is a risk of tumor development if these are integrated into genomic DNA. Therefore, regenerative medicine using mRNA, which poses no risk of genome insertion, has attracted attention. In this review, the author summarizes techniques for synthesizing mRNA and its application in regenerative medicine.

## 1. Introduction

Messenger RNA (mRNA) is produced in living organisms by transcription from genomic DNA, and proteins are produced based on the sequence information from mRNA. With the recent spread of coronavirus disease 2019 (COVID-19), attempts to apply mRNA as a medicine have attracted attention [1]. In addition to being used as a vaccine against cancer and viral and bacterial infections [2], it is also expected that mRNA will be used as a drug for protein supplementation therapy and tissue regeneration to restore function, enabling the generation of missing proteins in genetic diseases [3]. The research and development of mRNA therapeutics have been of interest for a long period of time, but these efforts have dramatically progressed since the COVID-19 pandemic [4].

**Figure 1:**
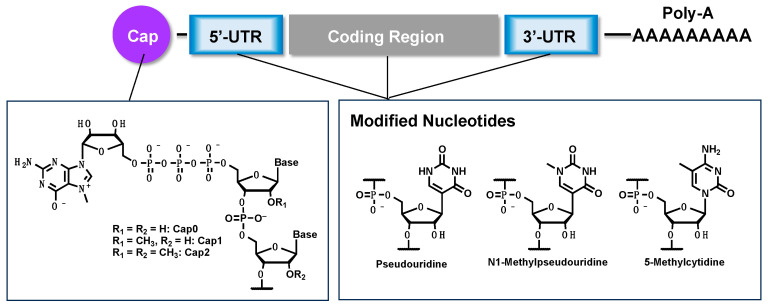
Structure and established chemical modifications of mRNA. mRNA consists of several domains, including 5′-cap, 5′-UTR, a coding region, 3′-UTR, and a poly-A tail. The 5′-cap structure is especially important for the initiation of the translation of mRNA. Modified nucleobases such as pseudouridine, N1-methyl pseudouridine, and 5-methylcytidine are generally introduced to the hole position of mRNA to reduce immunogenicity.

mRNA is composed of several regions, including a 5′ cap structure, a 5′-untranslated region (UTR), a protein-coding region, a 3′-UTR, and a poly-A tail (Figure 1) [5]. The 5′ cap structure is a characteristic structure present at the 5′ end of mRNA, in which 7-methyl guanosine is linked via a 5′-5′ triphosphate bond [6]. The 5′ cap structure was discovered by Furuichi et al. in Japan, and various cap structures have been reported, such as Cap0, Cap1, and Cap2, depending on the presence or absence of 2′-*O*-methyl modification [7,8]. This structure is essential for practical, therapeutic use of mRNA because it is involved in the intracellular stability of mRNA, translation initiation, splicing, and innate immune responses. Although the 5′- and 3′-UTRs do not directly encode proteins, they possess the function of controlling the translation activity of mRNA [9,10,11]. For example, internal ribosome entry sites (IRESs) [12], which are currently being actively studied, play an important role in recruiting ribosomes and initiating translation. The poly-A tail is involved in mRNA stability and initiating translation [13]. Understanding the function of the structural components of mRNA is important for designing mRNA that can be applied to cell reprogramming and differentiation tools.

In October 2023, Karikó and Weissman received the Nobel Prize in Physiology or Medicine for their important discoveries regarding the development of mRNA vaccines [14]. They discovered that mRNA therapeutics containing the natural uridine base showed a very high inflammatory response, whereas mRNA therapeutics in which the uridine base was converted to pseudouridine or N1-methylpseudouridine suppressed the inflammatory response (Figure 1). This made it possible to apply mRNA to practical medicine for the first time, representing a great contribution to humanity [15]. It has been suggested that mRNA therapeutics could apply, not only to vaccines, but also to protein supplementation therapy and regenerative medicine. In this paper, the author discusses the current progress of mRNA therapeutics development and research toward its use in regenerative medicine.

## 2. Synthesis of mRNA

The two main mRNA synthesis methods are post-capping using the vaccinia capping enzyme (VCE) and in vitro co-transcription [6]. The capping method using VCE is widely used to prepare mRNA. VCE is a capping enzyme derived from the vaccinia virus, which uses 7-methylguanosine 5′-triphosphate to add a cap structure to the 5′ end of transcriptionally synthesized RNA [16]. VCE exhibits three enzyme activities—RNA triphosphatase (RTPase), guanylyltransferase (GTase), and guanine methyltransferase (G-N7 MTase)—and can assemble the Cap0 structure. After the Cap0 structure is constructed by VCE, the Cap1 structure can be produced by 2′-*O*-MTase. The capping efficiency when using VCE is very high, but RNA sequence dependency is present. The COVID-19 mRNA vaccine developed by Moderna (mRNA-1273) is synthesized using the VCE approach. More recently, Ohno et al. focused on post-capping methods using VCE and investigated the introduction of various chemically modified cap structures [17]. They investigated the acceptability of such chemical modifications and their effects on translational activities and decapping resistance. According to their results, ribose 2′,3′-modification can be used for enzymatic capping, and modified-cap mRNA showed better decapping resistance and translational activity.

**Figure 2:**
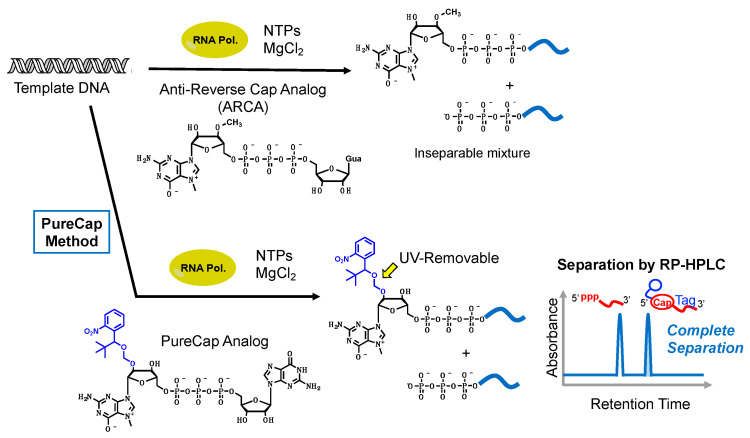
Methods of synthesizing mRNA using co-transcriptional cap analogs. In general in vitro co-transcriptional capping, an inseparable mixture of ARCA-capped and 5′-triphosphate RNAs is produced. Utilizing the PureCap method, 5′-capped RNA can be isolated by reverse-phase HPLC.

The co-transcriptional capping method is also used for practical mRNA preparation. BioNTech’s COVID-19 mRNA vaccines (BNT161b1 and BNT162b2) are produced by applying the co-transcriptional capping method. In the in vitro co-transcription capping method, a cap analog called ARCA [18], or CleanCap [19], is added to a transcription reaction solution with nucleoside 5′-triphosphates (NTPs), and the transcription reaction is carried out by RNA polymerases in the presence of template DNA. The transcription reaction is initiated from the cap analogs, yielding 5′-capped mRNA. However, the cap analogs and guanosine 5′-triphosphate (GTP) are competitively incorporated, resulting in a mixture of capped mRNA and undesirable uncapped mRNA. To overcome this problem, Inagaki et al. developed a new mRNA synthesis method, called the PureCap method, that can obtain completely capped mRNA [20]. In the PureCap method, an in vitro transcription reaction is performed using a novel cap analog, with an *o*-nitrobenzyl group functioning as a hydrophobic purification tag at the 7-methylguanosine moiety. The *o*-nitrobenzyl group is introduced only into the 5′-terminus of capped mRNA, making it significantly more hydrophobic than uncapped RNA. As a result, differences in retention time can be observed on reverse-phase high-performance liquid chromatography (RP-HPLC), making the clear separation of capped and uncapped mRNA possible (Figure 2). It has been shown that the PureCap method can produce highly purified mRNA with a Cap2-type structure, which had been difficult to synthesize using conventional methods. Although Cap0 and Cap1 mRNAs activate human cytosolic immune receptors, Cap2 mRNA can escape from binding with these immune receptors when administered to the cells. That means the Cap2 structure has a great benefit in reducing the immunogenicity of mRNA therapeutics. Additionally, completely capped mRNA can be used to study the structure–activity relationship (SAR) of mRNA 5′-cap structure diversity and protein translation activity. Furthermore, the translational activity of purified, fully capped mRNA was found to be more than 10 times higher than that of mRNA produced using conventional cap analogs. Inagaki et al. aim to contribute to mRNA therapeutics research by making it possible to produce highly pure mRNA using the PureCap method. Research progress related to mRNA synthesis is important in order to accelerate the development of mRNA therapeutics for cell regeneration therapy.

## 3. Key Technologies for mRNA Therapeutics

An early discovery regarding mRNA vaccine development was a synthesis demonstration by Krieg and Melton et al. in 1984 using a virus-derived RNA synthase [21]. Furthermore, in 1989, Malone et al. reported on mRNA mixed with lipid droplets inserted into frog embryos, suggesting the possibility of externally adding mRNA as a drug [22]. However, it has been recognized that synthetic mRNA is generally unstable in serum and difficult to apply as a medicine or vaccine. This instability has been overcome with the discovery of delivery techniques using lipid nanoparticles (LNPs) composed of phospholipids, cholesterol, ionized lipids, and PEG lipids. Currently, LNP technology is indispensable in the development of mRNA therapeutics. LNPs formulate mRNA and protect it against nuclease digestion [23], and the mRNA formulated by LNPs is incorporated into the cells by the endocytosis mechanism. This technology was developed primarily by Cullis et al. Since the 1990s, they have been working on developing the technology to use LNPs to deliver short (antisense) oligonucleotides that control gene expression in cells [24]. Several oligonucleotides have been approved as LNP therapeutic agents for genetic diseases [25]. In 2012, Cullis et al. started experimenting with applying LNP technology to mRNA delivery. In 2012, Geall et al. successfully prepared the first LNPs encapsulating an mRNA vaccine [26]. LNPs developed in this way are used in current COVID-19 vaccines. The development of LNP technology has made it possible to stably administer mRNA into cells. However, LNPs taken into cells by endocytosis reside within the endosomes. Therefore, it is necessary to release the mRNA from within the endosome, but the endosomal escape efficiency is generally only about 2% at most [27]. Furthermore, although delivery to the liver and spleen has been achieved, delivery to other tissues is difficult, and it is necessary to develop LNPs with tissue specificity. To expand the tissue available to restore the function by mRNA-based regenerative medicine, further research and development of LNP technology is important for the medicinal application of mRNA, and many researchers are involved in this effort [28].

Although mRNA therapeutics research has progressed with the development of LNPs, the expression of inflammatory responses when mRNA is administered to cells has also been noted as a serious problem. Karikó and Weissman received the 2023 Nobel Prize in Physiology or Medicine, and their co-workers succeeded in solving this problem. Starting in the 1990s, they refined Malone’s protocol and were able to demonstrate the expression of therapeutic proteins in cells [29,30]. However, in 1997, when they were developing an mRNA vaccine against HIV and administering synthetic mRNA to mice, a severe inflammatory response occurred [31]. It was determined that the reason for this phenomenon was that immune sensor receptors, including Toll-like receptors, which are nucleic acid receptors present in the cytoplasm, recognized the administered synthetic mRNA as non-self RNA [32]. Then, in 2005, they discovered that the inflammatory response could be suppressed by converting the uridine in mRNA to a base-modified nucleoside called pseudouridine [33]. By replacing uridine 5′-triphosphate with pseudouridine 5′-triphosphate during in vitro transcriptional RNA synthesis, pseudouridine modification could be introduced into the entire mRNA [15]. In fact, in Moderna’s COVID-19 vaccine, all of the mRNA contains modified bases.

On the other hand, the development of mRNA therapeutics that do not use modified mRNA is also attracting attention. Hertlein et al. hypothesized that by adding the appropriate 5′ cap structure to unmodified mRNA and removing all impurities, they should be able to create mRNA therapeutics that would be as effective as modified mRNA. In 2016, Hertlein and Anderson et al. showed that what matters is the quality of the RNA, and that unmodified mRNA exhibits greater activity than pseudouridine-modified mRNA if the mRNA is highly purified [34]. Our group reported that mRNA synthesized using the PureCap method showed a lower inflammatory response and higher translational activity than conventional mRNA [18]. In this way, the development of mRNA vaccines involves the substantial development of chemical technologies. Delivery, modified base introduction, and high-purity mRNA production methods are important, not only for mRNA vaccines, but also for regenerative medicine, and these technologies are currently being applied. In the following sections, the author discusses the application of mRNA technology for cell reprogramming and differentiation tools toward mRNA-based regenerative medicine.

## 4. mRNA-Based Protein Supplementation for Regenerative Medicine

**Figure 3:**
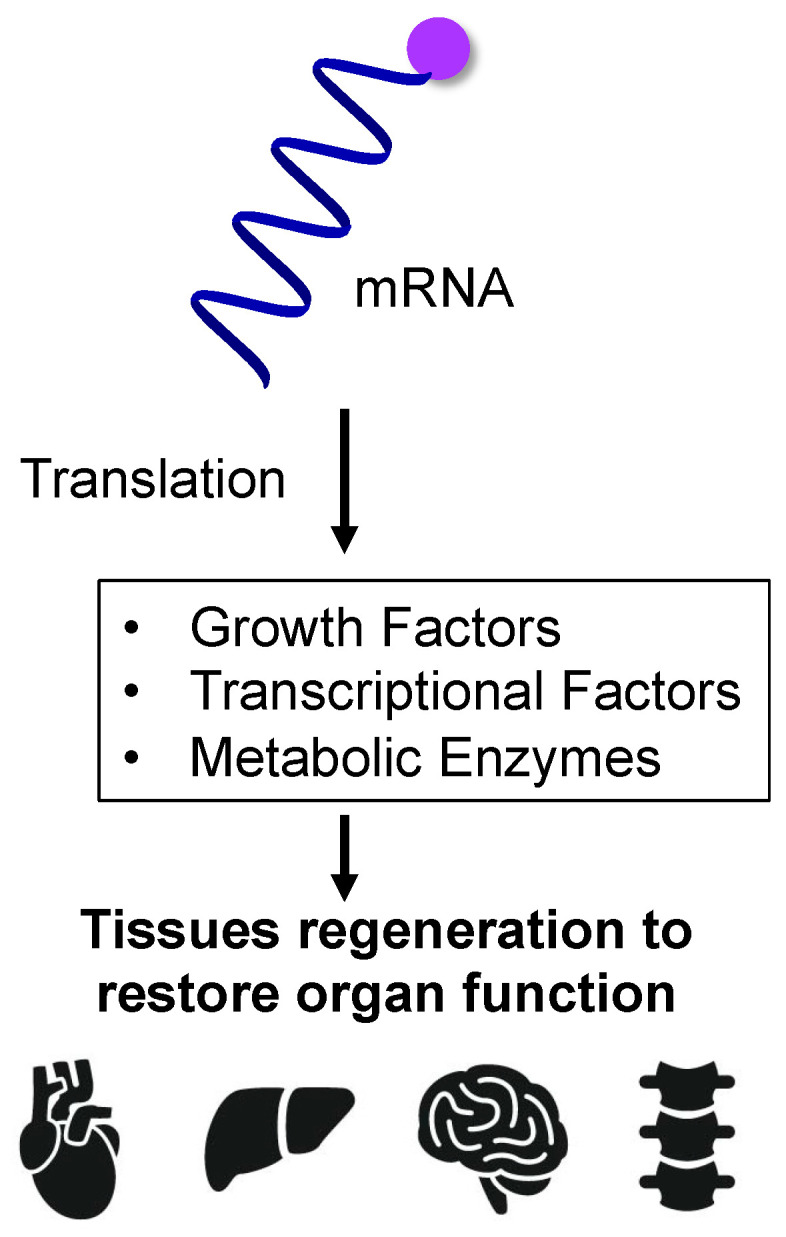
mRNA-induced tissue regeneration to restore organ function. Injecting mRNA encoding growth factors, transcriptional factors, and metabolic enzymes into specific tissue cells can restore the function of genetically deficient tissues.

Regenerative medicine is a treatment modality that restores function by regenerating tissues and organs lost due to accident or disease, and cell transplantation treatment using stem cells is a known form of this modality [35]. However, regenerative medicine requires a special cell culture facility, leading to higher medical costs, the risk of mutations occurring during cell culture, and slow supply speeds based on the time required for culture [36,37,38]. “Stem cells” is a general term for cells that have both self-renewal ability and multipotency and include somatic stem cells (adult stem cells, tissue stem cells), embryonic stem (ES) cells, induced pluripotent stem cells (iPSCs), etc. Somatic hepatocytes are cells that can modify and regenerate tissues by differentiating into new cells. ES cells are created by collecting cells from fertilized eggs that are in the process of becoming embryos and are taken from fertilized eggs that were not used for infertility treatment [39]. iPSCs, developed by Yamanaka et al. in 2006 [40,41], are stem cells that have been artificially returned to an undifferentiated state by incorporating multiple genes into mature somatic cells. In 2014, these became the first such cells in the world to undergo clinical research. In these treatments, the patient’s stem cells are differentiated outside the body into cells with the desired function, cultured, and then transplanted into the patient’s tissues to restore that function [42]. By using the patient’s cells, rejection after transplantation can be suppressed, but not all cells can be used. Additionally, it has been pointed out that it may be difficult for cells to proliferate and maintain in vitro due to limitations in their ability to proliferate. Therefore, regenerative medicine using mRNA is attracting attention [43]. Therapeutic effects are expected to be obtained by injecting mRNA into cells to express proteins, leading to the regeneration of lost tissue (Figure 3).

Examples of regenerative medicine using mRNA drugs include treatment for heart failure and fractures. Moderna is actively working on developing mRNA treatments for heart failure [44]. Heart failure is a disease that occurs when blood vessels in the heart become clogged or hardened, and one treatment option is to perform surgery to replace them with artificial blood vessels. In contrast, when an mRNA drug (AZD8601) encoding vascular endothelial growth factor A (VEGF-A) is directly administered to the patient’s myocardium, the VEGF-A is produced from the administered mRNA, promoting cardiac repair and regeneration [45]. Researchers at the Mayo Clinic are working on applications for fracture treatment. In a study using rats, researchers confirmed that administering mRNA encoding bone morphogenetic protein 2 (BMP-2), which promotes bone formation, to the fracture site promoted bone regeneration [46].

Most recently, Itaka et al. achieved rapid bone regeneration in bone defect animal models by administering mRNA encoding proteins that promote bone induction and angiogenesis [47]. They synthesized mRNA expressing osteoinductive transcription factor (Runx2), a therapeutic protein that exhibits bone regeneration effects, and vascular endothelial growth factor (VEGF), a secreted protein that plays a role in angiogenesis. These mRNAs were administered to undifferentiated osteoblasts, and their ability to induce osteogenic differentiation was evaluated. Even when Runx2 mRNA and VEGF mRNA were administered alone, the expression of bone differentiation markers (osteopontin, osteocalcin, etc.) was increased, suggesting that both Runx2 and VEGF can induce bone differentiation. When they were administered simultaneously, an even higher expression of osteogenic differentiation markers was observed, suggesting that the two act synergistically to induce the osteogenic differentiation of cells. Next, the bone regeneration effects of these mRNAs were verified using rats, in which a 4 mm diameter hole was formed in the jawbone. Better bone regeneration was achieved in the group with the defect compared to the no-treatment group and the control mRNA administration group. Consistent with the results obtained using cells, the most vigorous bone regeneration was observed in the group that received both in combination. Furthermore, histological evaluation suggested that VEGF-mRNA plays a role in both angiogenesis in bone defects and osteoinduction, and when combined with Runx2 mRNA, the two act synergistically.

Although some results have been reported in applied research on regenerative medicine using mRNA, some challenges remain [48,49,50,51,52,53,54,55,56,57,58]. Only short peptide fragments and proteins can be expressed by mRNA, and tissue regeneration involves not only peptides and proteins, but multiple other factors as well. Linking expressed peptides and proteins to tissue regeneration requires a deeper understanding of the tissue regeneration mechanism itself. In this section, the author focused on the introduction of protein supplementation therapy using mRNA. In the following section, the author discusses key examples of the applications for cell reprogramming using mRNA.

## 5. mRNA for Cell Reprogramming

ES cells are created by collecting cells from a fertilized egg that is in the process of becoming an embryo, and there are ethical issues involved [59]. On the other hand, iPSCs are cells with ES cell-like pluripotency and proliferation ability, established by introducing several types of factors into somatic cells and culturing them [60]. Unlike ES cells, which require early embryos, iPSCs can be created from any somatic cells, so there are no ethical issues or concerns about immune rejection. iPSCs can be differentiated into cells of almost any tissue or organ, so they are expected to have a wide range of applications [61]. The creation of models of damaged tissue/organ-like disease using iPSCs that can be used to examine the activity and safety of drug candidates is a good example. To generate iPSCs, reprogramming technology is required to initialize the epigenetic modification of the differentiated cells. iPSCs were first developed by Yamanaka et al. [40]. They discovered four genes, called Yamanaka factors (Oct4, Sox2, Klf4, and c-Myc), and injected them into fibroblasts using retroviral vectors to induce cell reprogramming. However, with retrovirus-based techniques, there is the possibility that the transgene will integrate into nuclear genomic DNA. Regarding the Yamanaka factors, there is concern that if Klf4 and c-Myc, which are involved in carcinogenesis, are accidentally inserted into genomic DNA, there is a risk of disease onset and tumor formation [62,63].

Since then, various techniques for creating iPSCs have been developed (Table 1). For example, several methods for introducing plasmid vectors and proteins are known, and although these are safer than the viral vectors, their cell introduction and reprogramming efficiency are insufficient [64]. Other reprogramming techniques using small molecules have been reported, such as ACTH 1-24 peptide (fragment of adrenocorticotropic hormone) [65], A83-01 (selective inhibitor of activin receptor-like kinase) [66], CHIR99021 (inhibitor of lycogen synthase kinase 3β) [67], SU5402 (FGF receptor inhibitor) [68], DAPT (inhibitor of γ-secretase) [69], LDN193189 (inhibitor of bone morphogenetic protein) [70], PD0325901 (selective inhibitor of MEK/MAPKK) [71,72], SB431542 (activin receptor-like kinase inhibitor) [73,74], SU5402 (tyrosine kinase inhibitor specific to fibroblast growth factor receptor) [75], and thiazovivin (improves the survival rate of human ES cells against trypsin treatment) [76]. Although reprogramming methods using such small molecules are extremely simple and innovative, it is necessary to confirm the required dosage and the presence or absence of cytotoxicity [77,78,79]. Furthermore, it is difficult to determine whether existing small-molecule reprogramming can cover all applications. It is necessary to expand the types of proteins and receptors that can be targeted and to search for further compounds. MicroRNA-induced reprogramming from somatic cells by injecting members of the mir-302 family (mir-302a, 302b, 302c, 302d, pre-microRNA cluster) is also reported in animal models [80]. The mir-302 family is highly expressed in slowly proliferating human ES cells, and rapidly decreases as the cells differentiate and proliferate. Reprogramming using microRNA is an effective method, but the only microRNAs that have been found to be involved in reprogramming are mir-302 [80], mir-372 [81], the miR-17-92 cluster [82], mir-19 [83], mir-524 [84], mir-371 [85], and mir-31 [86]; thus, in the future, it is necessary to explore the applicability of various microRNAs [87].

**Figure 4:**
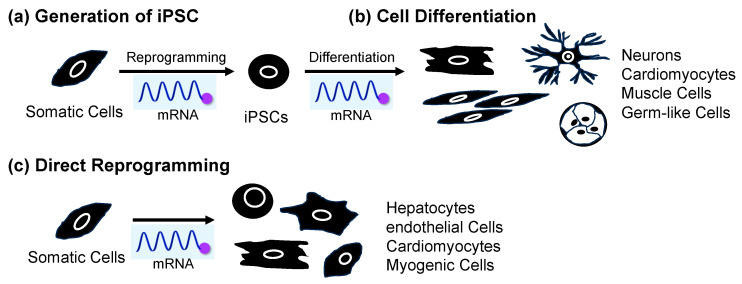
Application of mRNA for (**a**) cell reprogramming to produce iPSCs from somatic cells, (**b**) differentiation from iPSCs, and (**c**) direct reprogramming from somatic cells.

A reprogramming method using mRNA has been developed in recent years (Figure 4a) [88,89,90,91,92,93,94,95,96,97,98,99,100,101,102,103,104,105,106,107]. Similar to mRNA vaccines, mRNA reprogramming creates iPSCs by introducing mRNA containing genetic information to create cell reprogramming factors in cells and expressed target proteins. Compared to the retrovirus method of delivering DNA encoding reprogramming factors, mRNA is unstable within the cells and degrades gradually, so it does not remain in the iPSCs [108]. As a result, mRNA does not cause mutations in genomic DNA, and there is no risk of tumor development. It is also known that the reprogramming efficiency is higher compared to those of existing methods using viral vectors [92]. In reprogramming using mRNA, as in using mRNA as a vaccine, the introduction of chemical modifications is recommended [109,110,111]. Following the awarding of this year’s Nobel Prize in Physiology or Medicine, the use of chemically modified mRNA in COVID-19 vaccines has been attracting increased attention [15,34], but the application of chemically modified mRNA to cell reprogramming has also been considered since around 2010 [92].

LNP formulations similar to mRNA vaccines, electroporation, and liposomes have also been reported as cellular introduction methods. The mRNA used for cell reprogramming is loaded with 5-methylcytosine, pseudouridine, and 5′-cap structure [109]. In addition to OCT4, SOX2, KLF4, and c-MYC, LIN28 is commonly added as a reprogramming factor encoded by mRNA. Examples of mRNA-mediated reprogramming that have been reported to date include somatic cells such as fibroblasts [90,92,112,113,114,115], adipose-derived stem cells (ADSCs) [116], bone marrow-derived mesenchymal stem cells (BMSCs) [117], and amniotic fluid stem cells [118]. On the other hand, there are challenges in creating iPSCs using mRNA from blood cells, which are generally used to create iPSCs because they are easy to culture. mRNA needs to be injected every day due to its biological instability. Blood cells are resistant to cationic lipids [119], so lipofection cannot be used, and electroporation is the method of choice, but multiple courses of electroporation increase the risk of cell death [120]. Therefore, it is necessary to improve the intracellular stability of mRNA to ensure sustainable protein expression and reduce the number of administrations. Increased mRNA stability is important, but multiple turnovers of mRNA are found in cancer cells. Therefore, the signaling pathway change in cancer development induced by carcinogenic properties due to mRNA stabilization should be carefully investigated. Although there have been some successful cases of reprogramming using mRNA, there are limitations to its applicability, and further research and development are required.

A recent successful example of mRNA-induced reprogramming is the establishment of iPSCs derived from Alzheimer’s disease patients. In 2022, Supakul et al. established iPSCs for patients with mild Alzheimer’s disease using an iPSC establishment kit sold by ReproCELL, a biotech company [121]. They succeeded in establishing iPSCs from cells collected from a patient’s urine by administering an mRNA cocktail by lipofection. To date, most iPSCs have been produced from fibroblasts found in the skin or blood. Since urine is easier to collect than skin or blood, it is expected that it will become easier to generate iPSCs from patients with diseases, as well as from children, from whom it was previously difficult to collect samples [122]. Research using the generated iPSCs is thought to provide clues to solving social problems associated with aging, such as the increasing number of patients with dementia. In the future, when more examples of reprogramming using mRNA accumulate, it is expected that this will lead to the elucidation of the mechanisms of the development of various diseases and their application to therapeutic research. In addition to cell reprogramming, cell differentiation is also an important technique in regenerative medicine. The application of mRNA for cell differentiation is discussed in the following section.

## 6. mRNA-Induced Cell Differentiation from iPSCs

Applying iPSCs to regenerative medicine also requires technology to induce differentiation of reprogrammed iPSCs. There are three general methods for inducing differentiation of iPSCs into target tissue cells. The first is to prepare a cell culture medium containing a combination of various cell growth factors, cell differentiation factors, and small-molecule drugs and culture pluripotent stem cells in this medium [123]. In many cases, cells are differentiated by exposing them to different culture solutions sequentially. The second method is to create clusters or aggregates of pluripotent stem cells, which allows the cells to change and interact with each other within the clusters (self-organization) and differentiate into various types of cells [124,125]. These methods require multiple steps, so it takes time for the cells to differentiate into the desired cells, and it is necessary to confirm whether the cells are the same as the original cells existing in the body. The third method takes advantage of the fact that genes involved in transcriptional regulation determine the differentiation state of cells, and it induces differentiation by activating these genes in pluripotent stem cells [126,127,128]. This method directly manipulates transcriptional regulatory factors that determine the differentiation state of the cells, resulting in rapid differentiation. However, since it requires genome editing technology, such as the clustered regularly interspaced short palindromic repeats (CRISPR)/CRISPR-associated protein 9 (CRISPR/Cas9) system, there is a risk of cancer or malfunction due to the introduction of off-target mutations that cleave and edit sequences other than the target sequence [129,130]. On the other hand, this problem can be overcome by introducing mRNA encoding transcriptional regulatory factors. For this reason, research on differentiation induction using mRNA is currently attracting attention (Figure 4b).

As an example of the usefulness of differentiation induction using mRNA, a 2017 report showed that neurons could be rapidly generated from iPSCs derived from patients with Gaucher’s disease [131,132]. Glucocerebrosidase (GBA) is an enzyme that decomposes the glycolipid glycosylceramide, and Gaucher’s disease is caused by mutations in the GBA gene [133]. Glycolipids cannot be broken down, and the main symptoms include enlargement of the liver and spleen, anemia, and thrombocytopenia, but neurological symptoms can also appear, and the disease is classified into three types based, on the presence and severity of these symptoms (type I–III) [134]. Although type I Gaucher’s disease is relatively mild and does not cause neurological symptoms, it is known that the risk of developing Parkinson’s disease is extremely high (9% to 12%) in these patients as they get older [135]. It has been suggested that excessive accumulation of glycolipids in the brain influences the onset of Parkinson’s disease, but the mechanism is unknown. The relationship between glycolipid accumulation and α-synuclein was investigated using nerve cells generated from iPSCs derived from patients with type I Gaucher’s disease [131]. When the researchers synthesized mRNA encoding a transcription factor that promotes neural differentiation and administered it to patients, they were able to confirm glycolipid accumulation just 10 days after the start of differentiation. Although α-synuclein aggregation was not been detected at that point, it was found that the phosphorylation modification of α-synuclein involved in the process was enhanced, making it susceptible to neurodegeneration. In addition, by forcing the normal GBA gene to promote glycolipid degradation, α-synuclein phosphorylation could be suppressed, suggesting that glycolipid accumulation is directly involved in the onset of Parkinson’s disease. On the other hand, it was reported that with conventional neural differentiation techniques, glycolipids accumulated 60 days after the start of differentiation. With this method, it takes more than a month for neurons to form; therefore, it takes even longer to detect the phenotype. Thus, it was shown that the synthetic mRNA differentiation method not only enables short-term differentiation, but is also effective at rapidly reproducing disease-related phenotypes.

A recent research result is the successful creation of sperm stem cell precursors from iPSCs of the marmoset, an experimental primate [136]. The researchers induced marmoset iPSCs to become primordial germ-like cells (PGCLCs) by transfecting them with mRNA encoding the SOX17 gene, a master regulator of primordial germ cells. They transplanted the created marmoset PGCLCs under the kidney capsule of an immunodeficient mouse, and succeeded in developing pre-spermatogonia (sperm stem cell precursors). Gene expression and DNA methylation analysis revealed that this process nearly faithfully reproduced the in vivo germ cell development process (up to the newborn stage), and the newly developed method is useful for research on early germ cell development in primates. Sperm production from iPSCs has not yet been achieved in primates, including humans, and the process has only progressed to the production of pre-spermatogonia. The author hopes to advance the development toward sperm production, which will lead to the investigation of the causes of infertility and applications in reproductive medicine in the future. By combining mRNA-induced cell reprogramming and differentiation methods, the next research objective is direct reprogramming using mRNA. The application of mRNA for both cell reprogramming and differentiation, as well as direct re-programming, is discussed in the following section.

## 7. mRNA for Cell Reprogramming and Differentiation Induction and Direct Reprogramming without Passage through Pluripotent Stem Cells

Examples of mRNA being used for reprogramming and differentiation induction were discussed above. It is also possible to generate functional tissues by administering mRNA as a differentiation-inducing factor to iPSCs that have been reprogrammed and established with mRNA. That is, mRNA can act as both a reprogramming and a differentiation-inducing factor. In 2010, Warren et al. reported the transformation of fibroblasts into embryonic stem cells, which then differentiated into contractile muscle tissue, using modified mRNAs [92]. They synthesized the mRNA encoding Yamanaka factors Oct4, Sox2, Klf4, and c-Myc. In this mRNA, cytidine was completely replaced with 5-methylcytidine, and uridine was completely replaced with pseudouridine. When the mRNA was administered to cells, immunostaining showed that the Yamanaka factors were expressed and localized in the nucleus. Furthermore, protein expression by this mRNA peaked 12 to 18 h after introduction and then rapidly decreased, indicating that it degraded within 10 h after administration and did not remain in the cells.

Researchers have also successfully reprogrammed somatic cells. A five-factor cocktail (KMOSL) containing four Yamanaka factors, plus mRNA encoding LIN28, was used in Detroit 551 (D551) cells, MRC-5 fetal fibroblasts, BJ neonatal fibroblasts, and primary cells from adult patients with cystic fibrosis. When the KMOSL–mRNA cocktail was introduced daily into four cultured skin-derived fibroblast-like cells (CF cells), many human ES cell-like colonies appeared, along with more than 10 iPSCs from each somatic cell line. Furthermore, the established iPSCs expressed OCT4, SOX2, NANOG, and hTERT, the Oct4 gene was demethylated, and pluripotency-related genes, including SOX2, REX1, NANOG, OCT4, LIN28, and DNMT3B, were observed. Transcripts were elevated to levels comparable to those of human ES cells, indicating that mRNA-reprogrammed iPSCs are more similar to human ES cells than to virus-generated iPSCs. In addition, the conversion of BJ fibroblasts introduced to the iPSCs using the 5-factor mRNA cocktail exhibited an efficiency of 2%, regardless of the presence or absence of the Rho-associated kinase (ROCK) inhibitor Y-27,632. It was found to be two orders of magnitude more efficient than conventional virus-based methods. Next, the researchers introduced KMOS-mRNA or KMOS retrovirus into dH1f fibroblasts in parallel, and found that ES cell-like colonies began to appear after 2 weeks in those into which mRNA had been introduced, and on day 16, transfection occurred. By the last day of transfection, there was an outgrowth of ES cell-like colonies, whereas when using the KMOS retrovirus, no ES cell-like colonies appeared by this time point, but only from day 24 after gene transfer. The efficiency of iPSC establishment, determined by counting the beginning of colony appearance and TRA-1-60 positive colonies, was 1.4% and 0.04% for KMOS mRNA and KMOS retrovirus, respectively, with KMOS mRNA being 36 times more efficient. Fibroblast growth factor (FGF) was removed from the medium of the iPSC line established using mRNA, serum was added, the medium was spread on a gelatin coat, and mRNA encoding the muscle differentiation-inducing MyOD gene was introduced. After an additional 3 days of culture under low serum conditions, myogenin and MyHC double-positive myotubes appeared, with high efficiency. These results indicate that mRNA directly differentiates pluripotent stem cells into terminally differentiated cells.

It is expected that induction into tissue cells via ES cells and iPSCs will be applied in regenerative medicine. However, there are concerns about the risk of tumor formation due to undifferentiated cells and the low engraftment efficiency of treatments using pluripotent stem cells [106]. Direct reprogramming is attracting attention as a reuse method to solve the problems of stem-cell-derived cell transplantation [137]. This is a method for directly producing desired cells from fibroblasts, etc., without using iPSCs, and it is possible to produce tissue in vivo by introducing genes into target sites. The concept of direct reprogramming was proposed in 1987. The first report identified MyoD as a master factor for skeletal muscle, and by forcing the expression of the MyoD gene in fibroblasts, the researchers succeeded in producing fibroblasts, which are the precursors of skeletal muscle [138]. In 2010, Ikeda et al. were able to coax fibroblasts into becoming beating heart muscle. Using retroviral vectors, they revealed that Gata4, Mef2c, and Tbx5 (GMT) genes are essential for direct myocardial reprogramming. When these three factors were introduced into fibroblasts, a cardiac muscle-specific gene expression pattern was observed, as well as the expression of cardiac muscle-specific structural proteins, such as α-actinin and cardiac troponin (cTnT), with a sarcomere structure [139].

Since then, efforts have been made to identify factors that promote direct reprogramming. In 2014, Muraoka et al. reported the use of microRNA as a factor to promote direct reprogramming of the heart muscle [140]. They revealed that adding miR-133 to GMT efficiently induced myocardium in a short period. The enhancement of direct reprogramming using lower-cost small molecules is also being investigated. In 2015, Zhao et al. hypothesized that fibroblast plasma maintenance mechanisms inhibit reprogramming into myocardium. By using small molecules that suppress the TGF-β and ROCK pathways, which promote fibrosis, they succeeded in improving the efficiency of guiding mouse fetal fibroblasts to myocardium [141]. Furthermore, in 2019, Muraoka et al. showed that diclofenac, a nonsteroidal anti-inflammatory drug, suppressed age-related inflammation, thereby improving the efficiency of direct reprogramming from adult mouse fibroblasts to myocardium, which was difficult to induce [142]. Cardiomyocyte induction, using only small molecules and without using any genes, has also been reported. In 2016, Cao et al. reported that by introducing nine small molecules, they could induce human fibroblasts to become functional heart muscle [143]. The advantage of this method is that it is safer and provides relative ease of control regarding the cell culture conditions because it does not use genes or viral vectors. On the other hand, direct reprogramming using mRNA, which offers less risk of gene insertion, is also attracting attention (Figure 4c).

In 2014, Simeonov et al. reported the direct reprogramming of human fibroblasts to hepatocyte-like cells using synthetic mRNA [144]. They confirmed the generation of hepatocyte-like cells by the lipofection of three types of mRNAs, consisting of HNF1A and two genes from FOXA1, FOXA3, and HNF4A, into human fibroblast cells in an optimized haptic growth medium. In 2017, Pham et al. achieved the direct reprogramming of endothelial progenitor cells from skin fibroblasts using the mRNA encoding ETV2 gene [145]. Endothelial progenitor cells are important for angiogenesis, but their abundance in the human body is limited. With the development of this technology, it is expected that it will be applied to autologous transplantation by administering mRNA to skin fibroblasts.

Only recently has research been conducted on direct reprogramming using mRNA. Several applied studies using model animals in regenerative medicine have been reported. In 2021, Kaur et al. demonstrated direct reprogramming from non-cardiomyocytes to cardiomyocytes by using mRNA encoding four cardiac reprogramming genes (Gated, Mef2c, Tbx5, and Hand2) and three reprogramming-helper genes (dominant-negative TGFb, dominant-negative Wnt8a, and acid ceramidase). Using a lineage-tracking model of acute myocardial infarction in mice, they administered an mRNA cocktail at the time of myocardial infarction and found that 25% of cardiomyocyte-like cells in the scarred area were reprogrammed. As a result, significant improvements in cardiac function, scar size, long-term survival rate, and capillary density were observed. Through this research, the author expects the development of safe and highly efficient regenerative drugs for ischemic diseases using mRNA [146]. In August 2023, Qabrat et al. demonstrated direct reprogramming of mouse fibroblasts to myogenic progenitor cells (iMPCs) by administering MyoD-expressing mRNA and small molecules promoting myoD expression (forskolin, a cyclic AMP agonist; RepSox, a TGF-β receptor inhibitor; and CHIR99210, a GSK3 inhibitor). The generated iMPCs were shown to express a series of myogenic stem cell markers and to differentiate into contractile myotubes. Furthermore, in a mouse model of Duchenne muscular dystrophy, iMPCs strongly engrafted into skeletal muscle and restored dystrophin expression in hundreds of myofibers [147].

To improve the efficiency of direct reprogramming using mRNA, it is important to innovate the technology for introducing mRNA into cells. In 2015, Lee et al. were able to induce cardiomyocyte cells from cardiac fibroblast cells in mice by adding polyarginine-fused heart-targeting peptide (CRPPR-R9) to lipofectamine, a common lipofection reagent. They administered mRNA encoding Gata4, Mef2c, and Tbx5 (GMT) genes for 2 weeks. They showed that by adding CRPPR-R9, the efficiency of intracellular introduction was approximately two times higher compared to the rates for conventional lipofection, and the resulting translational efficiency was confirmed to be approximately three times higher. In this way, the development of highly efficient delivery technology is expected to lead to the ability to promote direct reprogramming within the human body [97]. In the above sections, the application of mRNA for cell reprogramming, differentiation and direct reprogramming was introduced. The next topic is the application of mRNA, based on the analytical aspect, for the development of purification methods of iPSCs and iPSC-derived cells, which can contribute to the research and development of regenerative medicine.

## 8. mRNA-Based Purification Methods of iPSCs and iPSC-Derived Cells

**Figure 5:**
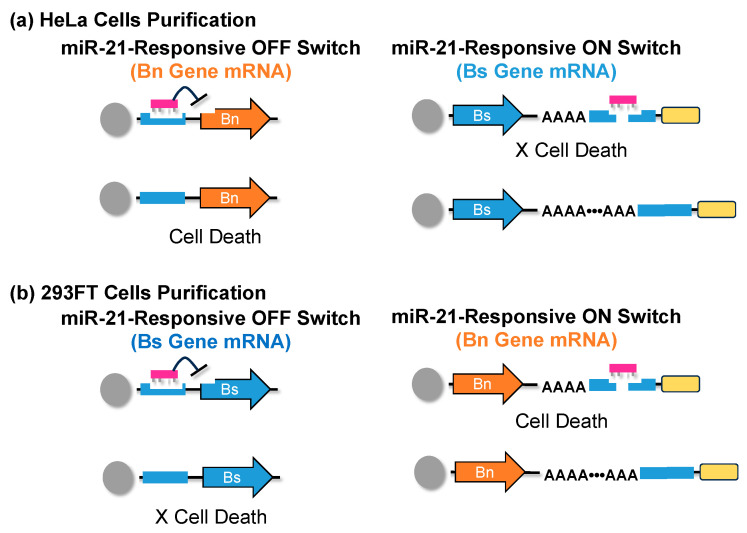
Purification of specific cells by microRNA-responsive mRNA switch system: (**a**) HeLa cells contain miR-21, which inhibits Bn expression of Bn, and express Bs to inhibit Bn; (**b**) 293FT cells do not contain miR-21; they express Bn and inhibit Bs.

iPSCs can differentiate into various cells, but after differentiation is induced, they also contain additional cells other than just the target cells. Therefore, the cells are sorted by identifying antigens on the cell surface using a flow cytometer [148,149]. However, when using a flow cytometer, there is the possibility that unintended cells or impurities could be mixed in during the operation of cell sorting, which is expensive, and it is difficult to prepare the necessary numbers of cells for transplantation. Also, it can take several hours to several days to obtain the required numbers of cells. Furthermore, it is often difficult to identify antigens specific to the target cells. iPSCs express tumor-specific microRNAs, and an mRNA switch technology, developed by Saito et al., that can identify these microRNAs and control gene expression shows that the purification of iPSCs is possible (Figure 5) [150].

This group synthesized mRNAs in which the genes for expressing barnase (Bn), a lethal ribonuclease that causes cell death, and barstar (Bs), a protein that inhibits Bn, were incorporated into the switch [151]. When purifying HeLa cells, they introduced the Bn gene into the microRNA response OFF switch that responds to miR-21, and the Bs gene into the microRNA response ON switch. HeLa cells that contain miR-21 [152] produce Bs protein that inhibits Bn due to the miRNA response ON switch, and cells that do not contain miR-21 produce Bn due to the microRNA response OFF switch, resulting in cell death (Figure 5a). When purifying 293FT cells, the Bs gene was introduced into the microRNA response OFF switch that responds to miR-21, and the Bn gene into the microRNA response ON switch. In 293FT cells that do not contain miR-21, the Bs protein that inhibits Bn is produced by the miRNA response OFF switch, and in HeLa cells that contain miR-21, Bn is produced by the microRNA response ON switch, resulting in cell death (Figure 5b). In addition, as a method to purify iPSCs established by reprogramming HeLa cells, the Bn gene was introduced into the microRNA response OFF switch that responds to miR-302a, and the Bs gene into the miRNA response ON switch. iPSCs that contain miR-302a produce Bs protein that inhibits Bn due to the miRNA response ON switch, and HeLa cells that do not contain miR-302a produce Bn due to the miRNA response OFF switch, resulting in cell death.

In this way, high-purity cell sorting was achieved without using a flow cytometer. This method can be applied to the purification of various types of cells. For example, researchers have also succeeded in purifying cardiomyocytes differentiated from iPSCs. This technology takes advantage of the characteristics of mRNA, which has a low risk of insertion into the genome, is easily degraded within cells, and does not linger, making it a highly safe and practical method for purifying various cell types for transplantation.

## 9. Conclusions

This paper outlines the progress in the design and methods for the synthesis of mRNA therapeutics and introduces the application of mRNA to vaccines, cell reprogramming, differentiation, and regenerative medicine. Overall, the technology described also appears to have clear potential for therapeutic applications in wound healing. The medical application of mRNA has been developed over many years of scientific and technological progress. The intracellular mRNA delivery technology and the introduction of chemically modified bases that exhibit anti-inflammatory effects, which are currently in practical use as vaccines, are important technologies not only for mRNA vaccines, but also for cell reprogramming and differentiation. Regenerative medicine using mRNA has significant advantages over conventional methods in terms of manufacturing cost, manufacturing speed, cell reprogramming efficiency, differentiation efficiency, safety, etc. Additionally, once the mRNA production method, delivery technology, and chemical modifications are established, a variety of applications can be realized by simply changing the introduced gene to match the target. For the future development of this field, it will be important to develop new science and technology related to mRNA therapeutics and practical mRNA production technology, and the author is also working on the development of these new technologies [18,153]. Applying mRNA to regenerative medicine requires the research and development of methods for separating differentiated and undifferentiated cells using the mRNA switch technology recently developed by Saito et al., as well as direct reprogramming that does not involve pluripotent stem cells [150]. Specifically, it is expected to be applied to therapeutic techniques that can regenerate tissue and restore dysfunction in intractable diseases such as neurodegenerative and fibrotic diseases that are difficult to treat using existing technologies. To this end, it is important to conduct further basic research on regenerative medicine using mRNA and to collect applicable examples.

## Figures and Tables

**Table 1:** Advantages and disadvantages of cell reprogramming strategies.

Reprogramming Method	Advantages	Disadvantages
Retroviral vectors	Well-investigated and established	Undesired transgene into nuclear genomic DNA
	High cellular introduction efficiency	Carcinogenesis and risk of tumor formation
Plasmid vectors	Low risk of genome insertion	Insufficient cellular introduction and reprogramming efficiency
Small molecules	Simple handling	Requires a relatively high dose
	Low cost	Necessary to consider dose-dependent cytotoxicity
	High cellular introduction efficiency	Difficult to cover all applications
microRNA	Fast reprogramming	Low physiological stability
	No risk of genome insertion	Fewer examples relative to other methods
mRNA	Fast reprogramming	Requires a more effective intracellular delivery method
	No risk of genome insertion or tumor development	Requires multiple injections (every day)
	High reprogramming efficiency	

## Data Availability

Not applicable.
